# Baseline characteristics and analysis of predictors of the Outcome of septic pulmonary embolism in children: a retrospective observational study

**DOI:** 10.1186/s12887-023-03998-z

**Published:** 2023-05-05

**Authors:** Rehab Elmeazawy, Doaa El Amrousy

**Affiliations:** Department of Pediatrics, Faculty of Medicine, Tanta University, Tanta, Egypt

**Keywords:** Septic pulmonary embolism, Children, CT chest, Cavitation, Outcome

## Abstract

**Background:**

Septic pulmonary embolism is a rare disease in children. We aimed to assess the clinical, microbiological, and radiological characteristics and outcomes of pediatric septic pulmonary embolism (SPE) and to identify any predictive factors for in-hospital mortality in patients with this unusual disease to enhance prognosis and treatment.

**Methods:**

A retrospective study to search the electronic medical records of children admitted to the pediatric pulmonology unit, Tanta University hospital with the diagnosis of SPE between January 2015 and June 2022.

**Results:**

Seventeen pediatric patients were identified; ten males and seven females with a mean age of 9.4 ± 5.2 years. The most common presenting complaints were fever and shortness of breath (n = 17) followed by chest pain (n = 9), pallor (n = 5), limb swelling (n = 4), and back pain (n = 1). Methicillin-resistant Staphylococcus aureus (MRSA) was the most common causative pathogen in nine patients. The most common extra-pulmonary septic foci were septic arthritis in five patients (29.4%), septic thrombophlebitis in four patients (23.5%), and infective endocarditis in two patients (11.8%). All patients exhibited wedge-shaped peripheral lesions and feeding vessel sign in CT chest, whereas bilateral diffuse lesions, nodular lesions, and cavitation were present in 94.1% of patients, pleural effusion was identified in 58.8% of patients, and pneumothorax was detected in 41.2% of patients. Fifteen patients improved and survived (88.2%), while two patients died (11.8%).

**Conclusion:**

Early diagnosis of SPE with vigorous early therapy is critical for a better outcome, including appropriate antibiotics and timely surgical interference to eradicate extra-pulmonary septic foci.

## Background

Septic pulmonary embolism (SPE) is a rare life-threatening disease in children, in which infected emboli from an extra-pulmonary septic focus reach the lung through the vascular system [1]. SPE presents clinically with non-specific respiratory symptoms such as fever, cough, chest pain, hemoptysis, and respiratory distress and is associated with high morbidity and mortality [2]. The most common causes of SPE are intravenous drug abuse, right-sided infective endocarditis, intravenous catheters, skin, and soft tissue infections [3, 4].

The most common microbiological organisms isolated in SPE are gram-positive cocci, including methicillin-susceptible Staphylococcus aureus and methicillin-resistant Staphylococcus aureus, and Klebsiella. However, few case reports have been published showing that SPE is caused by Fusobacterium, Pseudomonas aeruginosa, Acinetobacter, and fungal infections [5].

The diagnosis of SPE requires a high index of clinical suspicion and the documentation of specific radiological signs.

[4].The chest x-ray findings in SPE are non-specific and include unilateral or bilateral infiltrative lesions, diffuse cavitary lesions, pneumothorax, and pleural effusions [6]. Furthermore, more than half of the documented cases of SPE showed bilateral peripheral pulmonary nodules/cavitation on chest computed tomography (CT) [7].

Recently, with the era of the COVID-19 pandemic, the incidence of pulmonary embolism has increased. Autopsy studies in adults have identified large-vessel pulmonary artery thrombosis and/or embolism, small vessel pulmonary artery thrombosis, and deep vein thrombosis in the lower extremities and other sites [8].

Early diagnosis and management are crucial to improving the outcome of the patients. However, diagnosis of SPE remains difficult for clinicians because of its vague clinical manifestations and non-specific radiological findings. This work aimed to assess the clinical, microbiological, and radiological characteristics, and outcomes of SPE in children to facilitate early recognition, diagnosis, and treatment of this serious condition as well as to identify any predictive factors for in-hospital mortality in patients with this unusual disease to enhance prognosis and treatment.

## Methods

In this retrospective study, we searched medical records of children admitted to the pediatric pulmonology unit, Tanta University Hospital between the electronic January 2015 and June 2022 to identify cases of SPE. We could not obtain written informed consent from the patients’ guardians due to the retrospective nature of the study. The study was approved by the ethics committee of the Faculty of Medicine, Tanta University.

Inclusion criteria were age < 18 years and diagnosis of SPE. SPE was diagnosed according to Cook et al. criteria which include the following: CT chest scans showing diffuse nodular opacities or multifocal lung infiltrates compatible with septic embolism to the lung; the presence of a primary source of infection as a potential embolic source, exclusion of other causes of lung infiltrates, clinical and radiographic improvement after antibiotic therapy [9].

Exclusion criteria were malignancy, tuberculosis, non-septic pulmonary embolism, and interstitial lung diseases.

### Data collection

Using computer-based research, we identified thirteen children with SPE between January 2015 and June 2022 at the pediatric pulmonology unit, of Tanta University Hospital. We searched the medical records of the patients and the following data were extracted: age, sex, presenting symptoms, physical signs, the primary septic focus, duration of hospital stay, laboratory results, microbiological culture, radiological and echocardiographic findings, treatment received, and the outcome.

CT pulmonary angiography scans were evaluated for the presence of unilateral or bilateral lesions, peripheral pulmonary nodules, wedge-shaped lesions, feeding vessel signs, pulmonary consolidations, cavitations, pleural effusion, pneumothorax, and mediastinal lymphadenopathy. The outcome of the patients was based on survival or death.

### Statistical analysis

Data were collected, revised, and edited into a master table using Microsoft Excel 2013. Data were then revised, coded, and entered into the statistical package for social science (SPSS) version 23. Categorical data were presented as numbers and percentages, while quantitative data with normal distribution were presented as mean and standard deviation. To identify predictors of in-hospital mortality, logistic regression was used. The Hosmer Lemeshow test was used as a goodness-of-fit test to assess the fit of logistic regression models. P < 0.05 was considered statistically significant.

## Results

### Clinical characteristics

Seventeen pediatric patients were identified, ten male and seven female patients with a mean age of 9.4 ± 5.2 years (range 1–16 years). Table (1) shows the clinical characteristics of 17 children with SPE. The most common presenting complaints were fever and shortness of breath (n = 17) followed by chest pain (n = 9), pallor (n = 5), limb swelling (n = 4), and back pain (n = 1). The most common extra-pulmonary septic foci were septic arthritis (29.4%) followed by septic thrombophlebitis (23.5%), infective endocarditis (11.8%), soft tissue infection (11.8%), venous catheter-associated bloodstream infection (5.9%), vertebral osteomyelitis (5.9%), both soft tissue infection with venous catheter-associated infection (5.9%) and no focus of infection was detected in one patient (5.9%). The duration of the hospital stay ranged from 1 to 90 days. The duration of intravenous antibiotics ranged from 2 to 76 days, while the duration of oral antibiotics ranged from 0 to 30 days.

**Table 1:** Demographic, clinical, microbiological characteristics, and the outcome of SPE patients

Case No	Age (yr)/ sex	Presenting complaints	Septic focus	Microorganisms	Culture site	Pulmonary treatment	Extra-pulmonary treatment	PICU admission	Duration of hospital stay, (days)	Outcome
1	2.5/F	Fever, RD	Unknown	Staph.aureus	Blood	Antibiotics, oxygen	No	No	14	Survived
2	13/M	Fever, chest pain, RD	Septic hip arthritis	MRSA	Pleural fluid, Sputum, Pus	Antibiotics, oxygen, intercostal chest tubes	Drainage of hip joint twice	Yes	75	Survived
3	7.5/M	Fever, chest pain, RD	Septic hip arthritis	MRSA	Pericardial fluid and Blood,Pus	Antibiotics, oxygen	Drainage of hip joint- Pericardiocentesis	Yes	30	Survived
4	10/M	Fever, chest pain, RD, leg swelling	Septic thrombophlebitis of left leg	No growth	Blood	Antibiotics, oxygen	No	Yes	45	Survived
5	11/F	Fever, Chest pain	Soft tissue infection	Staph.aureus	Pleural fluid, Sputum, Blood	Antibiotics, oxygen	No	Yes	45	Survived
6	4/M	Fever, chest pain, RD, Rt leg swelling	Septic thrombophlebitis of right leg	MRSA	Blood	Antibiotics, oxygen, intercostal chest tubes	No	No	60	Survived
7	3/M	Fever, RD, Pallor	Infective endocarditis	Staph.aureus	Blood, Pleural fluid	Antibiotics, oxygen, intercostal chest tubes	Arrange for cardiac surgery	Yes	20	died
8	14/F	Fever, RD	Venous- catheter infection	MRSA	Blood, Central line culture	Antibiotics, oxygen	Hemodialysis	Yes	45	Survived
9	1.5/M	Fever, RD, Pallor, swelling of left elbow	Septic elbow arthritis	Staph.aureus	Blood, Pleural fluid	Antibiotics, oxygen, intercostal chest tubes	Drainage joint	Yes	30	Survived
10	15/F	Fever, chest pain, RD	Infective endocarditis	MRSA	Blood	Antibiotics, oxygen	Surgical removal of vegetations on TV with TV replacement	Yes	75	Survived
11	14/M	Fever, chest pain, RD, left leg swelling	Septic thrombophlebitis of left leg	MRSA	Blood	Antibiotics, oxygen, intercostal chest tubes	Steroids and immunosuppressive therapy	Yes	45	Survived
12	13/M	Fever, RD, left leg swelling	Septic thrombophlebitis of left leg	NA	NA	Antibiotics, oxygen	No	Yes	1	died
13	14/F	Fever, chest pain, RD, Back pain	L3 vertebral osteomyelitis	Klebsiella	Pleural fluid, BAL	Antibiotics, oxygen	No	Yes	60	Survived
14	16/F	Fever, chest pain, RD,pallor	Soft tissue infection	Staph.aureus	Blood	Antibiotics, oxygen	Steroids and immunosuppressive therapy	No	21	Survived
15	13/M	Fever, RD	Septic hip arthritis	MRSA	Blood	Antibiotics, oxygen	Drainage of joint	No	45	Survived
16	8/M	Fever, RD, chest pain	Septic knee arthritis	MRSA	Blood	Antibiotics, oxygen, intercostal chest tubes, Decortication	Drainage of joint	Yes	51	Survived
17	1/F	Fever, RD, Pallor	Soft tissue infection, venous- catheter infection	MRSA	Blood	Antibiotics, oxygen, intercostal chest tubes, Decortication	Drainage of skin abscess	Yes	55	Survived

F: female, M: male, RD: respiratory distress, PICU: pediatric intensive care unit, MRSA: methicillin-resistant Staphylococcus aureus, NA: not applicable, TV: tricuspid valve, BAL: bronchoalveolar lavage

### Microbiological results and antibiotics susceptibility

Methicillin-resistant Staphylococcus aureus (MRSA) was the most common causative pathogen (52.9%), followed by Methicillin-sensitive Staphylococcus aureus infection (MSSA) (29.4%), Klebsiella pneumoniae (5.9%), and no growth was detected in two patients (11.8%). Cultures of blood, sputum, pleural fluid, pericardial fluid, soft tissue, and bronchoalveolar lavage were performed for the patients as described in Table (2). All the patients received parenteral antibiotic therapy, according to our unit protocol till the results of culture and sensitivity appeared. Eleven patients (64.7%) showed ineffective empirical therapy due to complicated refractory bacteremia and had to change antibiotics after the results of cultures were obtained. The most effective antibiotic was vancomycin (82.4%), followed by linezolid and meropenem (41.2%).

**Table 2:** Baseline characteristics of 17 children with SPE

Variables			Frequency (N = 17)	Percentage %	Outcome	
					Survived (n = 15)	Died (n = 2)
**Age – Mean (Range) years**			9.4 (1–16)			
**Sex**	Male		10	58.8%	8	2
	Female		7	41.2%	7	0
**Clinical manifestations**	Fever & Shortness of breath		17	100%	15	2
	Chest pain		9	52.9%	9	0
	Pallor		5	29.4%	4	1
	Limb swelling		4	23.5%	3	1
	Back pain		1	5.9%	1	0
**Septic focus**	Unknown		1	5.9%	1	0
	Septic arthritis		5	29.4%	5	0
	Septic thrombophlebitis		4	23.5%	3	1
	Infective endocarditis		2	11.8%	1	1
	Central venous catheter		1	5.9%	1	0
	L3 vertebral osteomyelitis		1	5.9%	1	0
	Soft tissue infection		2	11.8%	2	0
	Soft tissue infection, Central venous catheter		1	5.9%	1	0
**Microorganism**	No growth		2	11.8%	1	1
	Staph.aureus		5	29.4%	4	1
	MRSA		9	52.9%	9	0
	Klebsiella pneumoniae		1	5.9%	1	0
**Ineffective empirical antibiotics**			11	64.7%	9	2
**Comorbid condition**	No		13	76.5%	11	2
	Inherited thrombophilia		1	5.9%	1	0
	Chronic kidney disease		1	5.9%	1	0
	Systemic lupus erythematosus		2	11.8%	2	0
**Pulmonary treatment**	Antibiotic, Oxygen		10	58.8%	9	1
	Antibiotic, Oxygen, chest tube		6	35.3%	5	1
	Antibiotic, Oxygen, chest tube, Decortication		1	5.9%	1	0
**Extrapulmonary treatment**	No		7	41.2%	5	2
	Drainage of joint		4	23.5%	4	0
	Drainage of joint and Pericardiocentesis		1	5.9%	1	0
	Cardiac surgery		1	5.9%	1	0
	Hemodialysis		1	5.9%	1	0
	Pulse steroids		2	11.8%	2	0
	Drainage of skin abscess		1	5.9%	1	0
**PICU admission**			12	70.5%	10	2
**Anticoagulants**			12	70.5%	10	2
**Joint U/S**		Joint effusion	6	35.3%	6	0
**CT findings**		Bilateral lesions	16	94.1%	14	2
		Nodular lesions	16	94.1%	14	2
		Cavitation	16	94.1%	14	2
		Pleural effusion	10	58.8%	9	1
		Pneumothorax	7	41.2%	6	1
		Consolidation	12	70.6%	10	2
**PCR for COVID-19**			1	5.9%	0	1

PICU: pediatric intensive care unit

### Radiological findings in SPE patients

Table (2) summarizes the radiographic features of patients with SPE.

#### Echocardiography findings

**Fig. 1:**
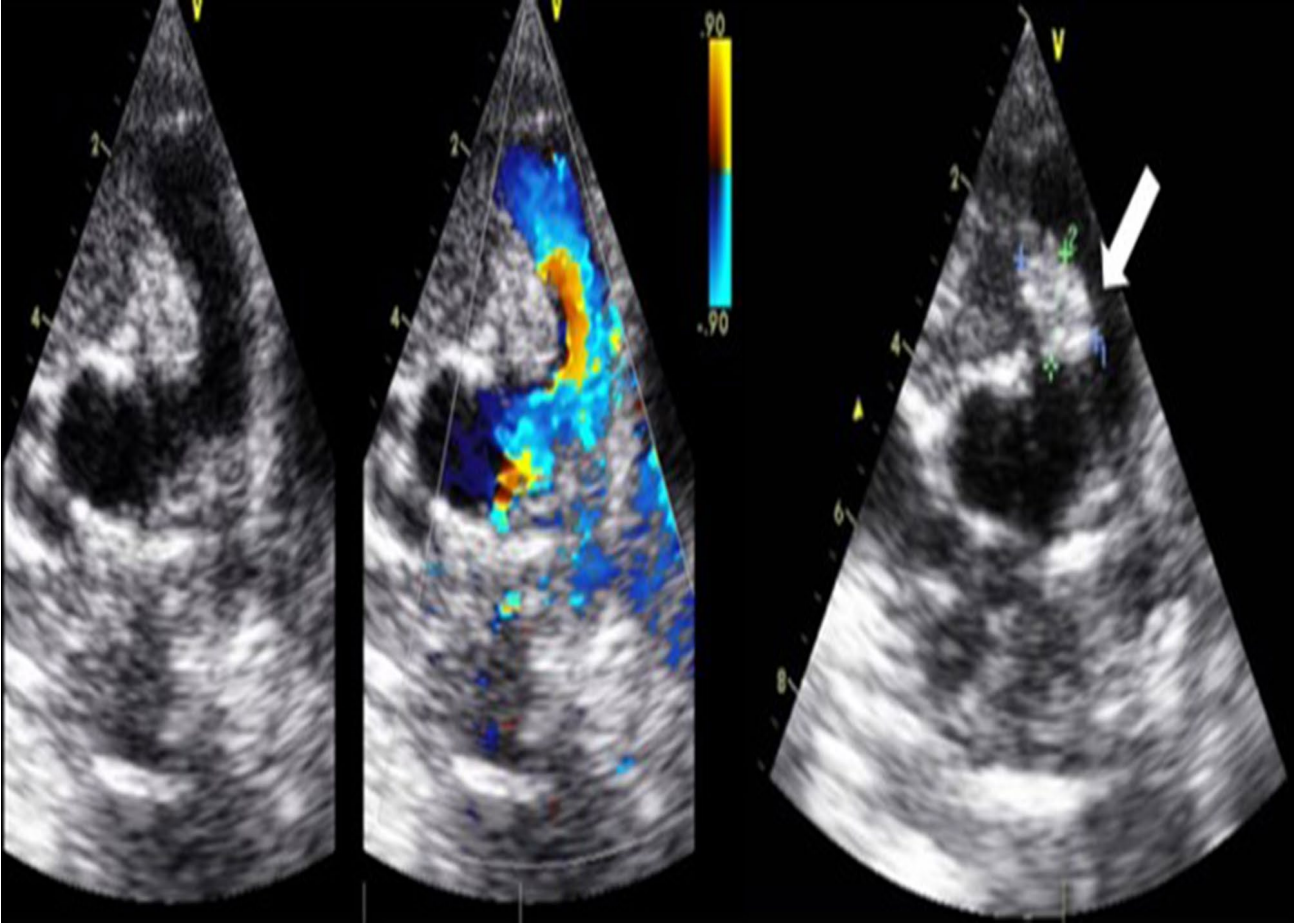
Transthoracic echocardiography showing a single large oval (11 × 13 mm) mass attached to the pulmonary valve (white arrow), it is highly mobile, homogenous, echo dense with smooth surface, no evidence of mural or intramyocardial extension. The mass causes a severe degree of pulmonary incompetence (G III/ IV) while not affecting forward pulmonary flow

Transthoracic echocardiography was performed in 16 patients, which showed tricuspid regurgitation in 70.6% of patients, mild-moderate elevated systolic pulmonary artery pressure (SPAP) was observed in (41.2%) of patients, right side cardiac vegetations were found in 3 patients (two patients on tricuspid valve and one patient had a large mobile mass on the pulmonary valve), and five patients had dilatation of the right side of the heart (Fig. 1).

#### Duplex findings

A duplex ultrasound scan was performed on 12 patients; six patients showed septic thrombophlebitis (4 patients had deep venous thrombosis (DVT), and the other six patients had a normal duplex scan.

#### Abdominal U/S

Abdominal ultrasound was performed on 15 patients. Abnormalities were identified in ten patients; four patients had hepatomegaly (23.5%), three patients had hepatosplenomegaly (17.6%), and three patients had bilateral nephropathy (17.6%).

#### Joint U/S

Six patients (35.3%) required joint ultrasound; three patients had left hip joint effusion, two patients had left knee effusion and one patient had left elbow effusion with surrounding edematous subcutaneous tissue.

#### CT chest findings

**Fig. 2:**
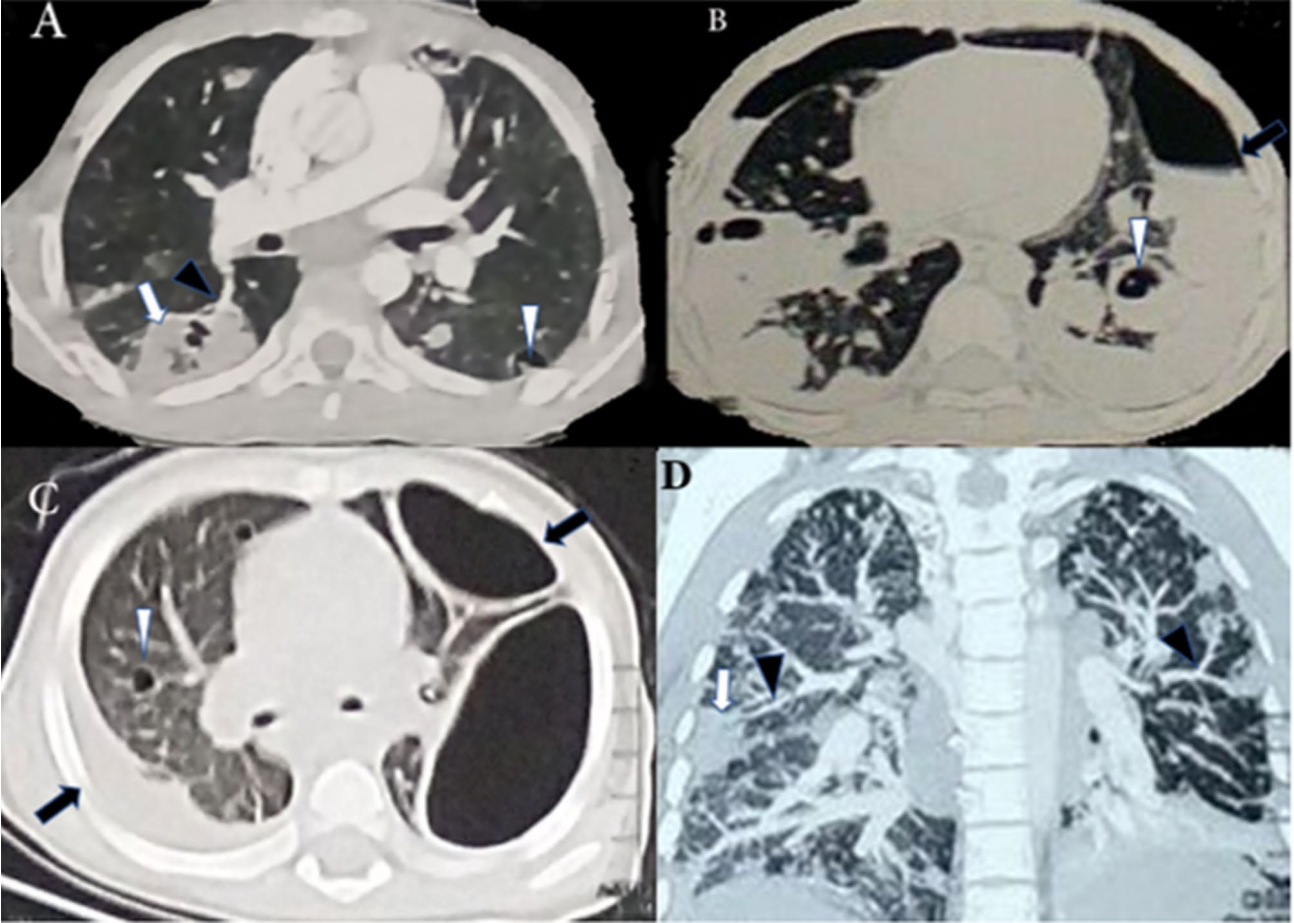
Chest CT revealed (A) right side peripheral wedge-shaped lesions (white arrow), feeding vessel sign (black arrowhead), and left side peripheral cavity (white arrowhead). (B) Bilateral pneumothorax (black arrow), and bilateral cavitary lesions (white arrowhead). (C) Left side encysted pneumothorax (black arrow), right multiple cavitary lesions (white arrowhead), and right side pleural effusion (black arrow). (D) Bilateral multiple nodular lesions (white arrow), and feeding vessel sign (black arrowhead)

Table (2) summarizes the CT chest findings in the 17 patients. All patients exhibited wedge-shaped peripheral lesions and feeding vessel signs, whereas bilateral diffuse lesions, nodular lesions, and cavitation were present in 16 (94.1%) patients. Pleural effusion was identified in 10 (58.8%) patients, and pneumothorax was detected in seven (41.2%) patients. In addition, consolidation was observed bilaterally in eleven (64.7%) patients, upper lobe consolidation in one patient (5.9%), and mediastinal lymphadenopathy in one patient (5.9%). Representative images of some radiological findings in children with SPE are shown in (Fig. 2).

### Laboratory findings

**Fig. 3:**
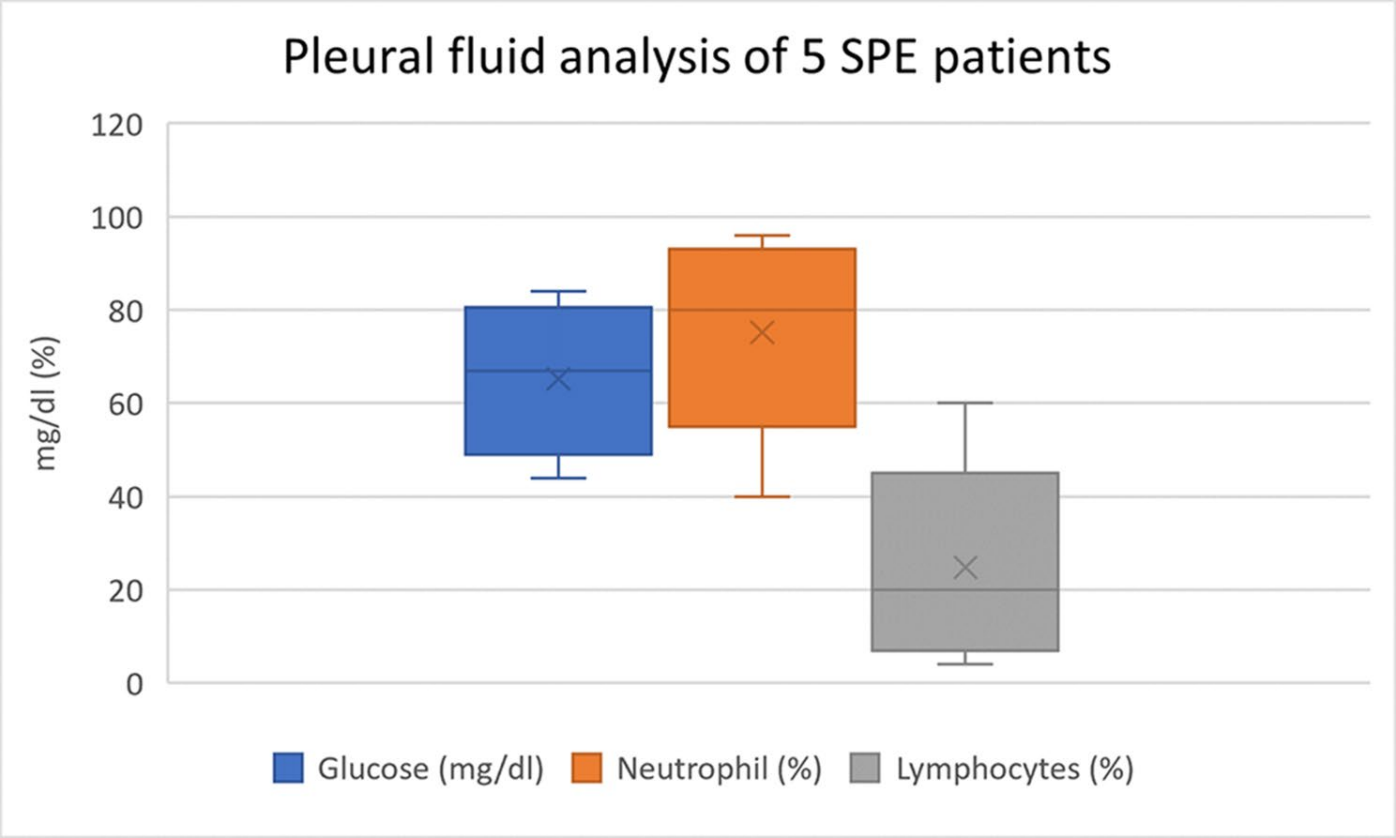
Box-and whisker plots showing pleural fluid analysis of 5 patients including pleural fluid glucose (mg/dl), pleural fluid neutrophils (%) and lymphocytes (%). The lower external bar represents the minimum value, the upper external bar represents the maximum value, the lower central bar represents the first quartile (Q1), the upper central bar represents the third quartile (Q3), the cross bar represents the median, the whiskers are the lines extending from the Q1 and the Q3 values to the minimum and maximum values, and the mean is indicated by ‘x’

Laboratory findings of the 17 patients with SPE were summarized in Table (3). Polymerase chain reaction (PCR) for factor V Leiden mutations was done in eleven (64.7%) patients; one patient was positive and ten (58.8%) patients were negative. A bone marrow aspiration test was done on eight patients; four (23.5%) patients showed toxic granulations while the other four patients showed normal results. As the study met the COVID-19 pandemic, we did PCR for the covid-19 virus in 6 (35.3%) patients; one patient showed positive PCR and the other 5 patients were negative. Serological tests for covid-19 were done in 5 patients with a positive IgM test in one (5.9%) patient, while IgG covid-19 was positive in 3 (17.6%) patients. Five patients underwent pleural fluid analysis. The protein content of the pleural fluid varied from 4.8 to 5.8 g/dl, whereas the LDH ranged from 301 to 7138 u/l. The total leucocytic count ranged from 500 to 35,000 cells/Cu mm. Please refer to (Fig. 3) for the remaining pleural fluid analysis results.

**Table 3:** Logistic regression analysis for predictors of SPE hospital mortality in children

Variables	Mean (Range)	Logistic regression analysis
		OR (95% CI)
Laboratory investigations		
Hb (gm/dl)	8.9 (5.6–12.6)	0.80 (0.29–2.22)
TLC (109/L)	15.5 (2.1–25.4)	1.01 (0.80–1.28)*
Platelet (109/L)	326.5 (51–973)	1.05 ( 0.99- 1.12)*
Lymphocytes (10^9^/l)	2.9 (0.9–7.2)	16.77 ( 0.51- 553.16)*
Platelet/ Lymphocyte ratio	115.1 (17.5–246.2)	1.02 (0.99- 1.05)*
Neutrophils (10^9^/l)	11.6 (0.9–20.2)	0.98 (0.76- 1.27)
Neutrophil/ Lymphocyte ratio	4.6 (0.8–12)	0.59 (0.30–1.14)
Monocytes (10^9^/l)	0.8 (0.1–1.8)	0.67 (0.02–20.92)
Monocyte/ Lymphocyte ratio	0.3 (0.1–0.9)	0.00 ( 0.00- 3.44)
CRP(mg/L)	130.7 (46–279)	1.01 (0.98–1.04)*
ESR 1st hr (mm)	97.5 (54–140)	1.02 ( 0.96–1.08)*
Blood urea (mg/dl)	43.9 (19–95)	0.95 (0.88–1.01)
Serum albumin (gm/dl)	3.1 (2–3.6)	6.96 (0.29- 164.47)*
D.dimer (ug/ml)	4.1 (0.78–10)	0.79 (0.44–1.43)
PT (sec)	18.1 (14–48)	0.84 (0.63–1.11)

Odds Ratios refer to the increase in one unit of each parameter

Hb: hemoglobin, TLC: total leucocytic count, CRP: C-reactive protein, ESR: erythrocyte sedimentation rate, PT: prothrombin time

### Treatment and outcome

#### Pulmonary treatment

Since the oxygen saturation in ambient air ranged from 50 to 97%, all seventeen patients got antibiotic medication along with oxygen delivered either through nasal prongs or facemasks, while seven patients required intercostal chest tube insertion either for pleural effusion or pneumothorax. Patients 2 and 6 required repeated chest tube insertion due to recurrent pneumothorax caused by repeated ruptured pneumatoceles. Patients 16 and 17 required lung decortication surgery.

#### Extra-pulmonary treatment

Patient 2 required local drainage of hip joint arthritis twice due to recollection of pus in the hip joint, while patient 3 needed local drainage of the hip joint with pericardiocentesis and pericardial tube insertion for 24 h. Patient 7 was arranged for cardiac surgery but unfortunately passed away suddenly from cardiac arrest mostly due to a flail pulmonary valve from large mobile vegetation. Patient 8 required frequent hemodialysis due to underlying end-stage renal disease. Patients 9, 15, and 16 needed local drainage of the affected joint while patient 10 required surgical removal of vegetations on the tricuspid valve with valve replacement. Patients 11 and 12 needed intravenous steroids with immunosuppressive therapy for underlying Systemic Lupus Erythematosus. Patient 17 required surgical drainage of a soft tissue abscess.

Twelve patients out of the 17 patients required pediatric intensive care unit (PICU) admission but none of them needed mechanical ventilation support. Anticoagulant therapy is controversial in the treatment of septic pulmonary embolism because of the theoretical risk of bleeding in the area of infected embolus and the lack of benefits. Twelve of our patients received anticoagulants in the form of low molecular-weight heparin (Enoxaparin). Regarding the outcome, fifteen patients survived without significant complications, while two patients died; one mostly due to large mobile pulmonary vegetation and the other one due to Covid-19 infection.

### Predictors for in-hospital mortality in pediatric patients with SPE

The rate of in-hospital mortality in our study was 11.8%. To determine the predictor factors, logistic regression and univariate analysis of laboratory parameters were done as shown in (Table 3).

## Discussion

SPE is a rare, serious complication of an infection in children that is difficult to diagnose partly due to non-specific presenting symptoms and partly due to the difficulty of identifying the primary source of infection. The process of septic pulmonary emboli starts with an extrapulmonary source of infection which leads to the extravasation of the pathogens, mostly bacteria into the systemic blood circulation. Once the organism reaches the bloodstream, it produces toxins and inflammatory mediators which results in thrombosis. The presence of a thrombus serves as a nidus for the proliferation and metastatic spread of the organism to the pulmonary circulation [10, 11].

In the present study, the most common presenting symptoms are fever, shortness of breath, chest pain, pallor, and limb swelling. Moreover, the most common pathogens were MRSA and other Staphylococcus aureus and these results agree with the results described before in previous literature [12, 13].

In a large cohort study done in China by Jiang J et al. on adult patients with SPE, they reported that skin and soft tissue infection (30.6%) was the most common foci of primary infection, followed by infective endocarditis (20.4%). This appears to be consistent with the causes of SPE in children. Wong et al. described in their study on pediatric patients that the commonest causes of septic PE were soft tissue and bone infections and they recommended that if the source is not clinically evident, a bone scan may identify a focus. In the present study, we emphasize the importance of septic arthritis and septic thrombophlebitis as the most common septic foci of infection [2, 14].

Interestingly, none of our patients seem to have Lemierre syndrome despite its known association with septic pulmonary embolism either as an explicit diagnosis or as suggested by jugular vein thrombosis of Fusobacterium that is contradictory to the results of other studies performed on adults [11, 15, 16]. However, Lemierre syndrome was considered in two of our patients, but Doppler ultrasonography on internal jugular vein did not reveal thrombosis. This could be due to the fact that the etiologic agents of SPE in the pediatric age group may differ from those of pediatric age group.

Additionally, Lemierre syndrome may be underestimated in the literature due to the fact that the diagnosis is often not posed without the evidence of Fusobacterium [17]. Similarly, Wong et al. [2] studied SPE in pediatrics and they had two patients with head infections (suppurative otitis media and bacterial conjunctivitis) but they did not refer to them as Lemierre syndrome because there were no positive cultures of Fusobacterium spp However, it is possible that other bacteria, especially some Streptococci and other non-Fusobacterium anaerobians can cause Lemierre syndrome as reported by a case reports [18].

Lastly, we cannot exclude that our sample did not include patients with primary signs of neck or pharyngeal infection but no initial suspicion of lung involvement, possibly causing cases of Lemierre syndrome to be underrepresented in our case series.

Typical radiographic features of SPE in CT chest include peripheral wedge-shaped densities of varying sizes that end with a feeding vessel sign. Other pulmonary findings include nodular lesions, cavitation, pleural effusion, pneumothorax, and sometimes reactive mediastinal lymphadenopathy. All these findings were present in our study. Lee et al. reported that peripheral nodules were the most common lesions (89.0%), while cavitation and feeding vessel sign was identified only in 10.4% and 6% of all the lesions respectively [19].

The most frequently prescribed antibiotics in our patients were vancomycin (82.4%), followed by linezolid and meropenem (41.2%). These results are in agreement with the results by Lin et al. and Wong et al. who found that vancomycin was the most effective antibiotic in their patients. In previous literature, some case reports used linezolid in the treatment of SPE in children with good results [2, 20–22].

The mortality rate for SPE in the present study was 11.8% (2 patients), while the survival rate was 88.2% (15 patients). The probable causes for mortality include large mobile pulmonary vegetation with a flail valve and the other one who died from severe Covid-19 infection. In Wong et al. study on 10 pediatric patients, there was no death and this may be attributed to their septic foci were non-cardiac lesions including soft tissue infections, bone infections, suppurative otitis media, and one patient with a catheter-related infection.

In a previous study done by Yusuf et al., the prognostic factors associated with an SPE high mortality rate in adult patients included low-level oxygen saturation and altered mental status. Oh et al. described the risk factors for mortality in adult patients with SPE were tachypnea and segmental or lobar consolidation on computed tomography. In the current study, the risk factor for mortality was Covid-19 infection which was incompatible with the previous studies that assessed the in-hospital mortality for SPE patients [23, 24].

Our study had several limitations. First, it is a retrospective single-center study which reduced the ability to find significant associations between variables. Second, we were unable to identify significant predictors of mortality due to the small sample size which is attributed to the rarity of the disease in children. Despite these limitations, our study provides valuable data to identify the clinical, radiological, and microbiologic characteristics of children with SPE.

## Conclusion

The present study confirms the findings of previous studies as regards the clinical presentation of SPE in children (fever, dyspnea, chest pain), the clinical applicability of the typical radiologic features (wedge-shaped peripheral lesions, feeding vessel sign, nodular lesions, and cavitations), and the potentially unfavorable prognosis. The primary sources of septic pulmonary emboli in children and the causative organisms may vary depending on the selected population and the ward of hospitalization. Appropriate antibiotic therapy, respiratory support, and early surgical intervention are essential for the treatment of patients with SPE.

## Data Availability

The datasets used and/or analyzed during the current study are available from the corresponding author upon reasonable request.

## List of Abbreviations

CT: Computed tomography.
IE: Infective endocarditis.
IQR: Interquartile range
MRSA: Methicillin-resistant staphylococcus aureus.
PCR: Polymerase chain reaction.
PICU: Pediatric intensive care unit
SPAP: Systolic pulmonary artery pressure
SPE: Septic pulmonary embolism.
SPSS: Statistical package for social science
